# Strengthening leaf physiological functioning and grain yield formation in heat-stressed wheat through potassium application

**DOI:** 10.3389/fpls.2022.1005773

**Published:** 2022-10-05

**Authors:** Muhammad Sarwar, Muhammad Farrukh Saleem, Hamza Maqsood, Najeeb Ullah, Aziz Khan, Muhammad Waqas, Nimra Sattar, Muhammad Tasneem, Xu Xu, Hu Zhangli, Yang Shuang

**Affiliations:** ^1^College of Life Sciences and Oceanography, Shenzhen University, Shenzhen, China; ^2^Department of Agronomy, University of Agriculture, Faisalabad, Pakistan; ^3^Faculty of Science, Universiti Brunei Darussalam, Gadong, Brunei; ^4^College of Agriculture Guangxi University, Nanning, China; ^5^College of Physics and Optoelectronic Engineering, Shenzhen University, Shenzhen, China

**Keywords:** wheat, potassium, foliar spray, grain filling, post-anthesis, heat stress, thermo-tolerance

## Abstract

Wheat crops are highly sensitive to high temperatures during their reproductive and grain-filling phases. We hypothesized that potassium could increase thermotolerance in wheat during grain filling by protecting cellular organelles, particularly chlorophyll, from heat injury. Two wheat genotypes, Ujala-16 (relatively heat tolerant) and Anaj-17 (relatively susceptible) were grown in pots and were submitted to 4 and 8 days of heat stress under polythene sheets 1 week after anthesis. One day before the onset of heat stress, 2% potassium (K) as K_2_SO_4_ was sprayed on all the plants. Flag leaves from both genotypes were collected after 4 and 8 days of heat stress. Leaf physiology changes were measured to quantify heat damage and to understand the K-induced recovery mechanism. The crop was harvested 125 days after sowing, and grain yield data were collected. Increasing duration of heat stress significantly impaired leaf physiology and grain yield of both studied wheat genotypes. Compared with control (under optimum temperature), 4 and 8 days heat-stressed plants produced 11 and 19% lesser grain yield per spike (averaged across genotypes and in the second years of study), respectively. Likewise, 4- and 8-days heat-stressed plants had 15 and 37% (averaged across genotypes and in the second years of study) lower flag leaf photosynthesis, respectively, compared with control plants. Across the genotypes, 8-days heat caused significantly more grain yield loss in Anaj-17 during the second year than in Ujala-16. Foliar K significantly restored leaf chlorophyll, Pn, Fv/Fm by reducing cellular membrane damage in the heat-stressed plants. This physiological recovery and activation of the plant defensive system by K under high-temperature stress protected the growth and grain development. For example, K_–_treated plants produced 19% higher 1,000 grain weight in 8 days of heat stress (across genotypes and in the second years of study) compared with water-treated plants under the hot environment of the respective thermal regime. Our study suggests that wheat performance under terminal heat stress can be improved through the exogenous application of K.

## Introduction

Global climate change and shifts in weather patterns have significantly increased the frequency of heat events during reproductive growth phases of wheat crops in many parts of the world (Ullah et al., 2020; Collins et al., 2022). Short episodes of heat during the early to mid-grain filling phase of wheat severely affect cell biochemistry, physiology, and overall grain yields (Ullah and Chenu, 2019; Shenoda et al., 2021). Yearly variations in grain yield recorded in many wheat-growing countries are associated with the rapid changes in temperature during grain filling (Djanaguiraman et al., 2020). For wheat crop, 21–23°C is considered the optimum temperature during grain filling (Porter and Gawith, 1999), and grain yield is reduced by 6% for each 1°C rise above this optimum temperature (Asseng et al., 2015). During the last 100 years, the global mean air temperature has increased by 0.8°C, and a further 2–4°C increase is projected for 2050 (IPCC, 2014). Numerous workers have reported that high temperature during grain filling reduces grain number and weight in wheat (El Sabagh et al., 2019; Ullah et al., 2019). Even a brief episode of heat (1 day) during early grain setting significantly diminishes wheat grain yield (Talukder et al., 2014).

Depending on severity and duration, post-anthesis heat stress limits carbohydrate formation and translocation toward developing grains (Sharkey, 2005). The reduction in growth and yield under a hot environment is associated with impaired physiological plant functioning. For example, high temperature in field crops disturbs the balance between reactive oxygen species (ROS) and the plant defensive system (Sarwar et al., 2017, 2018, 2019, 2021). Heat-induced cell membrane leakage during grain filling indicates oxidative damage to cellular organelles in wheat crops (Dias et al., 2010). Damage to leaf chlorophyll under hot environments results in poor photosynthesis (Pn), stomatal conductance (Gs), and chlorophyll fluorescence (Fv/Fm), and it inhibits the physiological functioning of wheat crops (Feng et al., 2014). Similarly, high temperature during grain-filling accelerates flag leaf senescence by 25% (Talukder et al., 2014; Djanaguiraman et al., 2020), although wheat genotypes with superior leaf greenness could sustain grain yield hot heat stress (Ullah et al., 2019; Mirosavljević et al., 2021). Similarly, genotypes with a capacity to sustain total soluble sugars (TSS), antioxidants, Pn, Gs are more adapted to heat stress (Sun et al., 2014; Nagar et al., 2015; Bala and Sikder, 2017).

Several approaches have been used for improving heat stress tolerance in crops, i.e., by developing heat-tolerant genotypes (Mondal et al., 2016), adjusting sowing dates (Saeed et al., 2017), and applying compatible solutes (Siddique et al., 2018), signaling molecules (Sarwar et al., 2021) growth regulators (Hu et al., 2016), and nutrients (Waraich et al., 2012; Sarwar et al., 2019). Foliar application of nutrients is one of the critical cultural practices for mitigating the adverse effects of heat stress on field crops (Raghunath et al., 2021). High temperature reduces nutrient uptake, and their utilization and partitioning in crops (Matías et al., 2021). Exogenous application of potassium increases leaf chlorophyll contents at grain filling of wheat. At the same time, higher leaf potassium in wheat and cotton crops under high-temperature stress also increases leaf chlorophyll contents (Zahoor et al., 2017).

Potassium, which is relatively immobile in soil, moves slowly by diffusion (Barber, 1984), and at grain filling of wheat, the K requirement is not met in calcarious soils even when the soil contains an adequate amount of potassium (Jifon and Lester, 2009). Despite the fact that K is relatively immobile in soil, it moves slowly through diffusion in the soil profile (Gäth et al., 1989), and even more slowly in soils with low cation exchange capacity (Wells et al., 1982). Under any abiotic stress, the plant expends its energy (ATP) to save its life (by producing compatible solutes and HSP), resulting in fewer ATPs available for roots to activate K^+^ uptake (Lester et al., 2005). When potassium uptake decreases, there is a deficiency within the root and leaf cells, and the concentration of reactive oxygen species in the cells increases (Shin and Schachtman, 2004). The Ca^++^ and K^+^ channels are affected by increased ROS production, and increased Ca^++^ concentration pushes potassium out of the cell (Pei et al., 2000). Foliar spray of K activates the defense system in heat-stressed wheat plants, protecting membranes from oxidative damage and strengthening physiological functioning (Shahid et al., 2020). For example, K foliar spray improved leaf Pn and carbon assimilation of heat-stressed wheat (Hassanein et al., 2013; Dhyani et al., 2016; Chaurasiya et al., 2018) by protecting chlorophyll contents and the membrane stability from oxidative damage (Rahman et al., 2014; Lv et al., 2017). Similarly, wheat genotypes sprayed with K at 70–80 days after sowing (DAS) exhibited significantly superior stay-green traits and produced higher grain yield under heat stress (Rahman et al., 2014).

Despite the positive impact of K on heat-stressed wheat, limited literature is available on how K protects developing wheat grains from short- and long-term heat spells. In this study, we quantified the impact of short and long episodes of heat on wheat on wheat grains and explored the specific role of K on grain yield formation. We hypothesized that (1) short and long-duration heat spells after anthesis impair plant physiology and reduce grain yield, and (2) potassium could increase thermotolerance in wheat during grain filling by protecting cellular organelles, particularly chlorophyll, from heat injury. The specific objectives of this study were: (1) to quantify the impact of short and long heat spells on wheat crops under controlled conditions, (2) to develop a technique to analyze heat damage, and (3) to explore the potential of K for alleviating wheat crop from heat damage.

## Materials and methods

**FIGURE 1:**
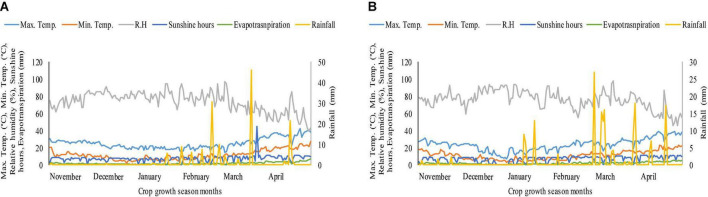
Weather conditions during crop growth period **(A)** 2018–2019 **(B)** 2019–2020.

The pot experiment was conducted in the wirehouse of the University of Agriculture, Faisalabad, Pakistan, during the Rabi season, 2018–2019, to observe the effects of heat durations and K on wheat genotypes 1 week after anthesis. The mean maximum temperature under polythene sheet for 4 and 8 days of heat stress, 1 week after anthesis, in 2019 was 29 and 28°C, respectively, while in 2020, the mean maximum temperature was 32 and 33°C for 4 and 8 days of heat stress, respectively, 1 week after anthesis (Supplementary Table 1). By making small holes in polythene sheets, relative humidity was kept between 70 and 80%. Water was applied to heat stress and ambient/natural environment pots on daily basis by measuring pan evaporation in order to maintain 100% field capacity in both heat stress and ambient/natural environment pots. Figure 1 depicts the weather conditions throughout the crop growing season during both years of study. The site is located at latitude 31°-26′ N, longitude 73°-06′ E, and altitude = 184.4 m. In this study, we used two wheat genotypes, Ujala-16 (relatively heat tolerant) and Anaj-17 (relatively susceptible). These genotypes were screened in a preliminary experiment based on relative cell injury, net photosynthetic rate, stomatal conductance, chlorophyll fluorescence, and seed yield (data not shown). The heat tolerant genotypes showed less cell injury, more photosynthetic rate, stomatal conductance chlorophyll fluorescence, and seed yield over the heat susceptible genotypes (data not shown). Both genotypes are commercially cultivated in wheat-growing areas of Pakistan. The seeds were collected from Wheat Research Institute, Ayub Agricultural Research Institute, Faisalabad, Pakistan. Soil was analyzed before the sowing of crop and had the properties as: organic matter 1.20 ± 0.025%; pH 8.2 ± 0.2, electrical conductivity (Ec-dS/m) 0.54 ± 0.013; available potassium 210 ± 5.9 ppm; available phosphorous 24.2 ± 0.6 ppm; available zinc 1.35 ± 0.035 ppm and available boron 0.7 ± 0.017 ppm. Each pot was filled with 18 kg soil with a peat and silt ratio of 3:1. Fifteen seeds were planted in each earthen pot (35 cm × 25 cm, length × width having 14.7 L capacity), which were thinned to 10 seedlings per pot after 1 week of germination and each pot received 2.24 g of urea (containing 46% nitrogen). Half of the nitrogen was applied at sowing, and the other half was applied 20 days after sowing. The pots were regularly watered to avoid heat drought complex.

### Imposition of heat stress and potassium

Seven days after anthesis, one set of plants (16 pots) was exposed to heat stress for 4 days, and the other set (16 pots) was subjected to heat stress for 8 days using a pierce polythene sheet (Saleem et al., 2018). The control set (16 pots) was kept in an ambient environment. Polythene sheet temperature was recorded daily using a digital multimeter (Digital Multimeter- 50,302) during heat imposition. The polythene sheet and the ambient temperatures are given in Table 1. Potassium (2%) was sprayed on half of the pots of each set 1 day before the start of the heat stress treatment. Potassium solution was mixed with a surfactant Tween-80 at 0.1% v/v (1 ml/L) and was applied early in the morning. In each pot, 80 ml of water was sprayed at 8 ml/s. Elemental sulfur was also sprayed in control pots of each set to account for the additional effects of sulfur. Polythene sheets were removed immediately after 4 and 8 days, and the data were collected for physiological and biochemical attributes. The experimental treatments were arranged under a completely randomized design (CRD) with split arrangements. There were four replications for each treatment with heat stress as the main factor while genotypes and foliar spray of K as sub-factors.

**TABLE 1:** Effect of optimal and stressful conditions of pots, potassium (K) spray and cultivars on superoxide dismutase (SOD U mg^–1^ protein), peroxidase (POD U mg^–1^ protein), catalase (CAT U mg^–1^ protein), and total soluble sugars (TSS mg/g of DW) of wheat flag leaves under 4 and 8 days of heat stress 1 week after anthesis.

Thermal regimes	Foliar spray of potassium	Cultivars	SOD		POD		CAT		TSS	
						
			2019	2020	2019	2020	2019	2020	2019	2020
Ambient temperature	Water spray	Ujala-16	71 ± 1.6 b	74 ± 1.81 a	34 ± 0.83 b	39 ± 0.92 c	80 ± 1.9 b	88 ± 2.1 b	22 ± 0.52 b	26 ± 0.57 b
		Anaj-17	66 ± 1.52 c	69 ± 1.71 ab	29 ± 0.68 c	32 ± 0.78 d	78 ± 1.9 bc	80 ± 1.9 b	20 ± 0.48 b	22 ± 0.53 c
	K (2%)	Ujala-16	77 ± 1.90 a	80 ± 1.9 a	40 ± 1.01 a	69 ± 1.72 a	90 ± 2.2 a	100 ± 2.4 a	26 ± 0.58 a	32 ± 0.66 a
		Anaj-17	70 ± 1.59 b	76 ± 1.85 a	33 ± 0.80 b	62 ± 1.42 b	82 ± 2.1 b	86 ± 2.1 b	25 ± 0.60 a	25 ± 0.61 b
2–3°C ± 2 rise in temperature for 4 days	Water spray	Ujala-16	83 ± 2.0 b	88 ± 2.0 c	50 ± 1.2 b	65 ± 1.61 c	88 ± 2.3 b	112 ± 2.7 c	25 ± 0.61 b	34 ± 0.82 c
		Anaj-17	72 ± 1.65 d	78 ± 1.90 d	35 ± 0.84 d	56 ± 1.41 d	82 ± 2.0 c	103 ± 2.6 d	22 ± 0.59 c	27 ± 0.69 d
	K (2%)	Ujala-16	91 ± 2.30 a	166 ± 4.12 a	65 ± 1.62 a	120 ± 3.01 a	100 ± 2.5 a	171 ± 4.2 a	32 ± 0.68 a	61 ± 1.47 a
		Anaj-17	77 ± 1.88 c	150 ± 3.70 b	43 ± 1.10 c	110 ± 2.61 b	88 ± 2.2 b	159 ± 3.9 b	26 ± 0.61 b	52 ± 2.30 b
2–3°C ± 2 rise in temperature for 8 days	Water spray	Ujala-16	89 ± 2.1 b	95 ± 2.32 c	59 ± 1.42 b	65 ± 1.60 c	100 ± 2.6 b	120 ± 3.1 c	31 ± 0.67 b	42 ± 1.02 c
		Anaj-17	76 ± 1.88 d	84 ± 2.11 d	43 ± 1.11 d	61 ± 1.41 d	88 ± 2.1 d	106 ± 2.7 d	25 ± 0.60 c	33 ± 0.79 d
	K (2%)	Ujala-16	100 ± 2.4 a	172 ± 4.20 b	79 ± 2.1 a	135 ± 3.32 a	115 ± 2.9 a	245 ± 6.1 a	40 ± 1.01 a	73 ± 1.61 a
		Anaj-17	83 ± 2.3 c	191 ± 4.71 a	53 ± 1.29 c	120 ± 3.10 b	95 ± 2.4 c	230 ± 5.9 b	30 ± 0.72 b	60 ± 1.45 b
**HSD**			**3.10**	**6.60**	**2.42**	**3.84**	**3.51**	**8.63**	**1.27**	1.67

Ujala-16 = Relatively heat tolerant, Anaj-17 = Relatively heat susceptible. Values are the means of four replications (*n* = 4) ± SE and variants possessing the same letters are not statistically significant at *P* < 0.01. HSD, honestly significant difference.

#### Leaf biochemical attributes

For biochemical analysis, three flag leaves were collected from each pot at the termination of heat stress treatment, i.e., 4 and 8 days. A composite sample was prepared from the leaves of each pot. A 0.5 g leaf sample was taken from the composite sample for the extraction of enzymes and reactive oxygen species. Superoxide dismutase (SOD) activity (U mg^–1^ protein) was measured by inhibiting nitro blue tetrazolium. A reaction mixture of 100 μl enzyme extract was used in an ELISA plate, and absorbance was recorded at 560 nm with an ELISA plate reader (Giannopolitis and Ries, 1977). Guaiacol oxidation procedure was used to quantify the leaf peroxidase (POD) activity (U mg^–1^ protein), and reaction mixtures along with 150 μl enzyme extract were taken in an ELISA plate, and enzyme quantity was measured at 470 nm (Liu et al., 2009). Hydrogen peroxide reactant was used to quantify the catalase (CAT) activity (U mg^–1^ protein) using (Liu et al., 2009) protocol. A reaction mixture containing 150 μl enzyme extract was used in an ELISA plate reader, and the absorbance was taken at 240 nm. The anthrone method measured total soluble sugars (TSS) (Yemm and Willis, 1954). Dry leaf tissues (0.5 g) were mixed with 5 ml of 95% ethanol. Alcoholic extract was preserved in the refrigerator, and 1/10th was mixed with 3 ml anthrone. The samples were placed in a boiling water bath for 10 min. Total soluble sugars were measured at 625 nm. Soluble sugars were quantified using glucose standard and measured as mg g^–1^ of DW of leaves. Leaf malondialdehyde (MDA) contents were measured according to the procedure described by Cakmak and Horst (1991). A 0.5 g leaf sample was homogenized with 3 ml of 0.1% (w/v) trichloroacetic acid (TCA), and the supernatant was extracted. A total of 3 ml of 20% TCA solution and 0.5% thiobarbituric acid were added to 0.5 ml of supernatant. A mixture, leaf extract, and a blank was added to an ELIZA plate, and the absorbance was measured at 532 and 600 nm.

#### Relative cell injury

Three flag leaves from each pot were collected at 12:00 p.m. after the termination of 4 and 8 days of heat stress and were averaged. A 10-mm diameter two leaf disks were obtained from both sides of flag leaves. Both leaf discs were washed 3-4 times with double distilled water and poured into test tubes having deionized water. One set of test tubes was treated in the water bath at 50°C for 1 h, while the other set of test tubes was maintained at room temperature at 25°C for an hour. The initial electrical conductivity (EC) was measured from both heat-treated and control test tubes at room temperature with an electrical conductivity meter (Model, Jenway 4510, Japan). After taking the initial EC, both test tubes were autoclaved (Model, HAU-85, Hirayam instruments, Tokyo, Japan) for 10 min at 0.1 MPa pressure. The final EC of both test tubes was determined at room temperature. Relative cell injury (RCI) was determined according to Sullivan (1972).


(1) $$\mathrm{RCI}\%=1-\frac{1-\left(\frac{\text{T}\,1}{\text{T}\,2}\right)}{1-\left(\frac{\text{C}\,1}{\text{C}\,2}\right)}\times 100$$

Where, C_1_ and C_2_ are the initial and final EC of control test tubes and T_1_ and T_2_ are the initial and final EC readings of heat-treated test tubes.

#### Photosynthetic parameters

Flag leaf gas exchange components and chlorophyll fluorescence (Fv/Fm) from three plants of each pot were measured immediately after removal 4 and 8 days heat stress in both experiments at 10:00 to 12:00 h and were averaged. An infrared gas analyzer (LCi Analyzer having Broad Head, Part Number LCi-002/B with Serial Number 32455) was used to determine the net photosynthetic rate (Pn, μ mol m^–2^ s^–1^) and stomatal conductance (Gs mol m^–2^ s^–1^) at three different developmental phases of the of wheat crop. These gas exchange characteristics were measured from flag leaves. Thylakoid membrane stability was assessed by measuring Fv/Fm using a Multi-mode chlorophyll fluorometer (OptiScience, UK with Serial Number 0729501) after 20 min dark adaptation of leaves. Maximum Fv/Fm was calculated as an indicator of plant stress (Prasad et al., 2008).

Three flag leaves from each pot and five flag leaves from each plot of both experiments were collected before sunset after removing heat stress to determine chlorophyll a/b contents. A total of 0.5 g sub-sample from each pot and plot leaves was taken and soaked overnight in 80% acetone. A blank with 80% acetone and leaves extracts of 1.5 μl were taken in an ELISA plate, and the absorbance was recorded at 645 and 663 nm wavelength in an ELISA plate reader for chlorophyll a and b contents (Arnon, 1949).

#### Flag leaf senescence and yield observations

Three flag leaves from each pot were tagged to measure the leaf senescence, yield per spike, and grain weight per spike at the end of 4- and 8-days of heat stress. Flag leaf senescence of selected plants was measured 1 day before and 7 days after heat stress. The senescence percentage of selected leaves was measured with a measuring scale, and the values were averaged. The crop was harvested 125 days after sowing, and the number of grains per spike (NGPS), grain weight per spike (GWPS-gram), seed yield per spike, seed weight per spike and 1,000 grain weight were recorded from the selected plants and were averaged.

#### Statistical analysis

A three-way analysis of variance was used to assess the effect of heat levels × foliar spray of K × genotypes under ambient and polythene sheet environments. Data at *P ≤ 0.05* were analyzed statistically using Fisher’s analysis of variance technique (Steel and Torrie, 1960). Tukey’s honestly significant difference (Tukey’s HSD) test was employed to compare the means at 1% using STATISTIX 10.1 software (Gomez and Gomez, 1984). Before running the combined ANOVA, a separate ANOVA was run for each factor. Graphs were drawn using MS Excel-2016, and the correlation matrix and the PCA analysis were performed in XL-STAT software.

## Results

### Grain yield and yield components under heat stress of polythene sheets

**FIGURE 2:**
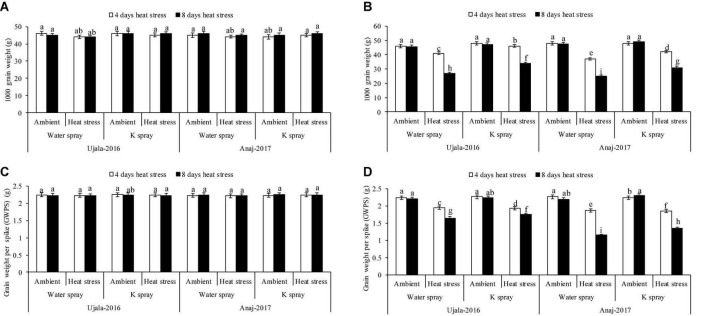
Impact of optimal, 4 and 8 days of heat stress (1 week after anthesis), potassium (K) spray and cultivars (heat × K × cultivars *P* < 0.01) on 1,000 grain weight **(A)** 2019 **(B)** 2020 and grain weight per spike **(C)** 2019 **(D)** 2020 of wheat. Values are the means of four replications (*n* = 4) ± SE and variants possessing the same letters are not statistically significant at *P* < 0.01.

**FIGURE 3:**
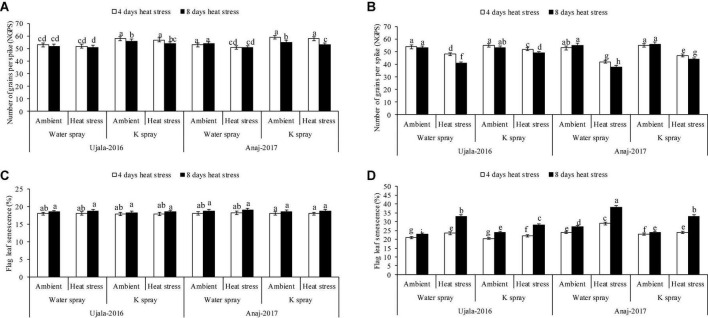
Impact of optimal, 4 and 8 days of heat stress (1 week after anthesis), potassium (K) spray and cultivars (heat × K × cultivars *P* < 0.01) on number of grains per spike **(A)** 2019 **(B)** 2020 and flag leaf senescence **(C)** 2019 **(D)** 2020 of wheat. Values are the means of four replications (*n* = 4) ± SE and variants possessing the same letters are not statistically significant at *P* < 0.01.

The wheat crop responded variably to the studied years. At 4 and 8 days of heat stress, 1 week after anthesis, the mean maximum temperature under polythene sheets was 29 and 28°C in 2019, while the temperature was 32 and 33°C in 2020. The mean maximum temperatures (both 4 and 8 days) in 2019 showed mild/non-significant effects on yield components, i.e., grain weight per spike, grain numbers per spike and 1,000 grain weight in both wheat genotypes. However, in 2020, the 4 hot days had a relatively lesser impact on yield components, but the 8 hot days (33°C ± 2) significantly reduced the grain yield components in both genotypes. In the second year of the study, for example, grain weight per spike, grain number per spike and 1,000 grain weight were reduced by 15, 11, and 14%, respectively, in 4 days of heat stress, and the decrease in three yield attributes was 34, 19, and 37% in 8 days of heat stress over the ambient/natural plants of respective thermal regimes (Figures 2, 3A,B).

Ujala-16 Heat-sensitive genotype (Anaj-17) and heat tolerant genotype (Ujala-16) produced 16% and 9% fewer grains per spike (NGPS), respectively, in 4 days of heat stress, while 26 and 15% fewer NGPS, respectively, in response to 8 hot days in the second year when compared with plants of ambient environments (Figures 3A,B). Similarly, in the second year, 1,000 grain weight of heat tolerant and heat susceptible genotypes was reduced by 30 and 41% in response to 8 hot days, respectively (Figures 2A,B). However, both genotypes produced a similar grain yield under ambient conditions in both study years.

Flag leaf senescence was increased in 4 and 8 days of heat stress in both genotypes in the second year of study, but Anaj-17 showed relatively more damage than Ujala-16 (Figures 3C,D). For example, 39 and 29% higher flag leaf senescence was observed in Anaj-17 and Ujala-16, respectively, in 8 days of heat stress over the ambient environment. Similarly, 4 days and 8 hot days resulted in 12 and 35% more flag leaf senescence (average across genotypes and the second year) than ambient plants.

Across genotypes, foliar K (2%) spray more effectively increased the grain yield and leaf greenness of the heat tolerant than of heat susceptible genotype under high-temperature stress; however, K had no effect in both genotypes under ambient environment (Figures 2, 3). Anaj-17Ujala-16Foliar spray of K increased 1,000 grain weight of Anaj-17 and Ujala-16 by 11 and 12% in the second years of study, respectively, under 8 days of heat stress, while 6 and 8% increase was observed in both genotypes under 4 days of heat stress over the non-K treated plants of the respective thermal regime (Figures 2A,B). Foliar spray of K also significantly reduced flag leaf senescence by 14 and 16% in Ujala-16 under 4 and 8 days of heat stress over Anaj-17 (during the second year) (Figures 3C,D). However, the effect was not significant under an ambient environment. Overall, wheat genotypes experienced 13% (averaged across genotypes) more grain losses in response to 8 days of heat stress over 4 days. 1,000 grain weight losses were 30% more in 8 than in 4 hot days (Figures 2A,B). Across the genotypes, 8 days of heat stress caused 34% more flag leaf senescence than 4 hot days. Overall, foliar spray of K (2%) increased 7 and 9% NGPS; 7 and 12% 1,000 grain weight and reduced flag leaf senescence by 8% and 10% across the genotypes under 4 and 8 days of heat stress, respectively.

### Leaf physiological functions in response to heat stress under polythene sheets

Across the genotypes and in the second year of study, Pn, Gs and Fv/Fm were reduced by 14, 10, and 11% in 4 days heat-stressed plants (32°C ± 2), while 24, 21, and 22% reduction were observed in 8 days heat-stressed plants (33°C ± 2) compared with plants of the ambient environment. Similarly, under high-temperature stress, leaf chlorophyll contents and leaf physiology were reduced more in heat susceptible genotypes than heat tolerant genotypes. During the first year of study, leaf physiological functions were not reduced prominently by 4 days (29°C ± 2 averaged across) and 8 days of heat stress (28°C ± 2 averaged across) when compared with the ambient environment. Exogenous application of K significantly restored the leaf physiological traits (Pn, Gs, Fv/Fm and chl a) of heat stressed plants across the 4 and 8 days of heat stress in the second year of study, with a relatively more recovery in heat tolerant genotype and under 8 days of heat stress (Ujala-16) (Tables 2, 3). Across the genotypes, Pn, Gs, Fv/Fm and Chl a contents of K-treated leaves were increased by 14, 10, 11, and 10% in 8 days of heat-stressed plants compared with their non-K treated leaves under respective heat environments (Tables 2, 3).

**TABLE 2:** Effect of optimal and stressful conditions of pots, potassium (K) spray, and cultivars on malondialdehyde (MDA nmol g^–1^ FW), relative cell injury (RCI%), net photosynthetic rate (Pn μmol m^–2^ s^–1^), and stomatal conductance (Gs m mol m^–2^ s^–1^) of wheat flag leaves under 4 and 8 days of heat stress 1 week after anthesis.

Thermal regimes	Foliar spray of potassium	Cultivars	MDA		RCI		Pn		Gs	
						
			2019	2020	2019	2020	2019	2020	2019	2020
Ambient temperature	Water spray	Ujala-16	0.55 ± 0.012 a	0.61 ± 0.014 b	43 ± 1.10 c	48 ± 1.18 b	30 ± 0.69 b	29 ± 0.69 b	0.91 ± 0.020 a	0.92 ± 0.020 a
		Anaj-17	0.57 ± 0.013 a	0.81 ± 0.018 a	50 ± 1.21 a	54 ± 1.31 a	30 ± 0.68 b	29 ± 0.68 b	0.92 ± 0.019 a	0.91 ± 0.021 a
	K (2%)	Ujala-16	0.50 ± 0.011 c	0.42 ± 0.011 c	35 ± 0.50 d	40 ± 1.07 c	31 ± 0.71 a	30 ± 0.69 a	0.93 ± 0.021 a	0.91 ± 0.019 a
		Anaj-17	0.54 ± 0.013 ab	0.63 ± 0.015 b	45 ± 1.14 b	50 ± 1.21 b	31 ± 0.70 a	30 ± 0.70 a	0.92 ± 0.022 a	0.91 ± 0.018 a
2–3°C ± 2 rise in temperature for 4 days	Water spray	Ujala-16	0.59 ± 0.014 c	1.05 ± 0.026 b	46 ± 1.21 c	65 ± 1.59 b	29 ± 0.68 b	25 ± 0.58 b	0.86 ± 0.016 b	0.81 ± 0.019 c
		Anaj-17	0.63 ± 0.012 a	1.41 ± 0.034 a	56 ± 1.38 a	88 ± 2.19 a	29 ± 0.69 b	24 ± 0.57 c	0.84 ± 0.021 b	0.77 ± 0.017 d
	K (2%)	Ujala-16	0.56 ± 0.013 d	0.75 ± 0.017 d	40 ± 0.97 d	52 ± 1.23 c	30 ± 0.70 a	28 ± 0.65 a	0.90 ± 0.019 a	0.88 ± 0.017 a
		Anaj-17	0.60 ± 0.014 b	0.87 ± 0.021 c	51 ± 1.22 b	66 ± 1.61 b	29 ± 0.68 b	25 ± 0.59 b	0.88 ± 0.018 a	0.83 ± 0.020 b
2–3°C ± 2 rise in temperature for 8 days	Water spray	Ujala-16	0.61 ± 0.015 c	1.40 ± 0.035 b	50 ± 1.20 a	82 ± 1.98 b	27 ± 0.66 b	22 ± 0.52 b	0.88 ± 0.017 a	0.73 ± 0.018 b
		Anaj-17	0.67 ± 0.017 a	1.69 ± 0.041 a	49 ± 1.19 a	92 ± 2.27 a	26 ± 0.60 b	20 ± 0.49 c	0.85 ± 0.022 b	0.64 ± 0.015 d
	K (2%)	Ujala-16	0.56 ± 0.013 d	0.85 ± 0.021 d	45 ± 1.12 c	57 ± 1.39 c	29 ± 0.67 a	26 ± 0.53 a	0.90 ± 0.020 a	0.81 ± 0.021 a
		Anaj-17	0.64 ± 0.016/b	0.92 ± 0.023 c	47 ± 1.17 b	61 ± 1.51 c	28 ± 0.66 a	22 ± 0.51 b	0.86 ± 0.015 ab	0.70 ± 0.016 c
**HSD**			**0.024**	**0.055**	**1.88**	**2.80**	**0.94**	**0.97**	**0.027**	**0.029**

Ujala-16 = Relatively heat tolerant, Anaj-17 = Relatively heat susceptible. Values are the means of four replications (*n* = 4) ± SE and variants possessing the same letters are not statistically significant at *P* < 0.01. HSD, honestly significant difference.

**TABLE 3:** Effect of optimal and stressful conditions of pots, potassium spray and cultivars on chlorophyll fluorescence (Fv/Fm) and chlorophyll contents (Chl a, b mg g^–1^ FW) of wheat flag leaves under 4 and 8 days of heat stress 1 week after anthesis.

Thermal regimes	Foliar spray of potassium (K)	Cultivars	Fv/Fm		Chl a		Chl b	
					
			2019	2020	2019	2020	2019	2020
Ambient temperature	Water spray	Ujala-16	0.94 ± 0.024 a	0.93 ± 0.022 a	1.75 ± 0.040 a	1.79 ± 0.043 a	0.23 ± 0.0049 a	0.27 ± 0.0054 c
		Anaj-17	0.92 ± 0.021 ab	0.90 ± 0.020 ab	1.73 ± 0.039 a	1.76 ± 0.042 a	0.24 ± 0.0050 a	0.25 ± 0.0052 d
	K (2%)	Ujala-16	0.95 ± 0.023 a	0.94 ± 0.023 a	1.77 ± 0.042 a	1.80 ± 0.044 a	0.22 ± 0.004 ab	0.31 ± 0.0074 a
		Anaj-17	0.96 ± 0.025 a	0.91 ± 0.021 ab	1.73 ± 0.041 a	1.78 ± 0.041 a	0.21 ± 0.0051 b	0.29 ± 0.0072 b
2–3°C ± 2 rise in temperature for 4 days	Water spray	Ujala-16	0.87 ± 0.019 a	0.80 ± 0.018 c	1.71 ± 0.040 a	1.59 ± 0.037 c	0.26 ± 0.0064 b	0.31 ± 0.0073 c
		Anaj-17	0.84 ± 0.018 ab	0.76 ± 0.017 d	1.67 ± 0.039 ab	1.53 ± 0.035 d	0.29 ± 0.0071 a	0.37 ± 0.0078 a
	K (2%)	Ujala-16	0.88 ± 0.020 a	0.87 ± 0.021 a	1.74 ± 0.042 a	1.69 ± 0.039 a	0.24 ± 0.0062 c	0.28 ± 0.007 d
		Anaj-17	0.86 ± 0.018 a	0.81 ± 0.016 b	1.69 ± 0.038 ab	1.63 ± 0.037 b	0.25 ± 0.0063 c	0.34 ± 0.008 b
2–3°C ± 2 rise in temperature for 8 days	Water spray	Ujala-16	0.83 ± 0.020 b	0.72 ± 0.015 c	1.65 ± 0.037 a	1.47 ± 0.032 c	0.31 ± 0.0076 a	0.45 ± 0.010 c
		Anaj-17	0.81 ± 0.017 b	0.64 ± 0.014 d	1.61 ± 0.038 ab	1.39 ± 0.030 d	0.34 ± 0.078 b	0.55 ± 0.013 a
	K (2%)	Ujala-16	0.87 ± 0.020 a	0.78 ± 0.018 a	1.70 ± 0.042 a	1.61 ± 0.037 a	0.27 ± 0.0065 c	0.36 ± 0.009 d
		Anaj-17	0.82 ± 0.018 b	0.73 ± 0.016 b	1.64 ± 0.034 ab	1.51 ± 0.036 b	0.30 ± 0.0077 d	0.47 ± 0.011 b
**HSD**			**0.034**	**0.030**	**0.055**	**0.058**	**0.011**	**0.018**

Ujala-16 = Relatively heat tolerant, Anaj-17 = Relatively heat susceptible. Values are the means of four replications (*n* = 4) ± SE and variants possessing the same letters are not statistically significant at *P* < 0.01. HSD, honestly significant difference.

Both 4 and 8 days of post-anthesis heat significantly increased lipid peroxidation (MDA) and relative cell injury (RCI) in both genotypes, but the more considerable damage was observed in 8 days of heat stress in Anaj-17 (heat susceptible genotype) during the second year of study. While during the first year of study, the effect of 4 and 8 days of heat stress was not observed as prominent on MDA and RCI. Foliar spray of K significantly reduced the MDA and RCI contents of both wheat genotypes under both 4 and 8 days of heat stress (Table 2). For example, K_–_treated plants had 34 and 23% lower MDA and RCI, respectively, under 4 days of heat stress, and 40 and 32% under 8 days of heat stress, respectively, compared with their respective non-K plants (averaged across genotypes and second year of study). Across the genotypes and years of study, K was relatively more effective in reducing MDA and RCI contents of Ujala-16 than of Anaj-17 under both 4 and 8 days of heat stress. Antioxidant enzymes (SOD, POD, CAT) and total soluble sugars (TSS) were increased by 61, 76, 54, and 66% (averaged across genotypes and second years of study) in 4 days of heat stress and 81, 89, 97, and 98% increase were observed in 8 days of heat stress, compared with plants of ambient environment of both stress regimes (Table 1). In general, a significantly more increase in antioxidant enzymes and total soluble sugars was measured in Ujala-16 than in Anaj-17 in both years of study. Antioxidant enzyme activities (SOD, POD, CAT) and total soluble sugars (TSS) were further stimulated in response to the foliar spray of K under both temperature regimes and across the genotypes. Further up-regulation was observed in Ujala-16 under both 4 and 8 days of heat stress. Averaged across the genotypes in second year of study, K-treated leaves had 1.02 folds, 1.03 folds, 1.10 folds and 0.77 folds higher SOD, POD, CAT and TSS contents, respectively, under 8 hot days compared with their respective water-treated leaves (Table 1). The correlation matrix shows that the number of grains per spike, grain weight per spike, 1,000 grain weight, leaf physiological traits and total soluble sugars were positively correlated with each other but negatively correlated with stress indicators, i.e., MDA, RCI, and flag leaf senescence (Table 4).

**TABLE 4:** Correlation matrix of malodialdehyde (MDA), relative cell injury (RCI), total soluble sugars (TSS), net photosynthetic rate (Pn), flag leaf senescence (FLS), number of grains per spike (NGPS), grain weight per spike (GWPS), and 1,000 grain weight (1,000 GW).

Variables	MDA	RCI	TSS	Pn	FLS	NGPS	GWPS	1,000 GW
MDA	**1**	0.9805	–0.8349	–0.9577	0.9254	–0.9395	–0.9153	–0.9582
RCI	0.9805	**1**	–0.8619	–0.9631	0.9347	–0.9429	–0.9188	–0.9349
TSS	–0.8349	–0.8619	**1**	0.9092	–0.9268	0.9581	0.9462	0.9079
Pn	–0.9577	–0.9631	0.9092	**1**	–0.9699	0.9841	0.9832	0.9840
FLS	0.9254	0.9347	–0.9268	–0.9699	**1**	–0.9755	–0.9680	–0.9591
NGPS	–0.9395	–0.9429	0.9581	0.9841	–0.9755	**1**	0.9923	0.9832
GWPS	–0.9153	–0.9188	0.9462	0.9832	–0.9680	0.9923	**1**	0.9774
1,000 GW	–0.9582	–0.9349	0.9079	0.9840	–0.9591	0.9832	0.9774	1

#### Principal component analysis

**FIGURE 4:**
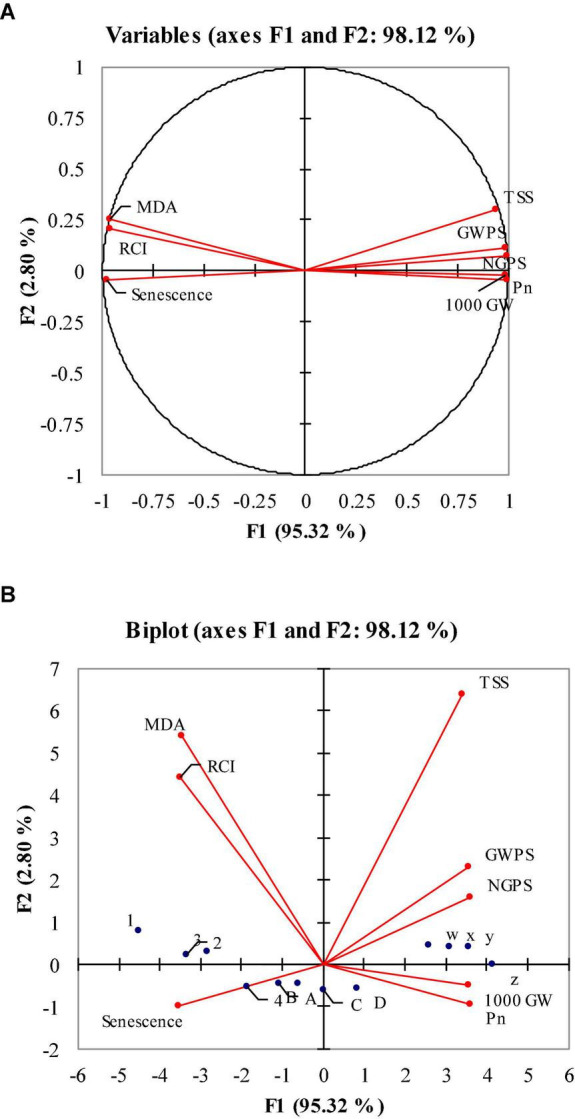
Principle component analysis (PCA) **(A,B)**: monoplot and biplot correlation of malondialdehyde (MDA) relative cell injury (RCI), net photosynthetic rate (Pn), flag leaf senescence, 1,000 grain weight (1,000 GW), grain weight per spike (GWPS) and number of grains per spike (NGPS) under optimal, 4 and 8 days of heat stress 1 week after anthesis of polythene sheet (averaged across both years of study).

Principal component analysis (PCA) was performed to estimate the relative effects of flag leaf senescence, MDA and RCI on grain yield, grain weight and TSS of heat-stressed wheat. PCA loading matrix shows a strong negative correlation of flag leaf senescence, MDA and RCI with NGPS, GWPS and 1,000 grains weight during post-anthesis heat stress. Malondialdehyde, relative cell injury and flag leaf senescence had a strong and positive relationship with each other, while 1,000 grain weight, Pn, GWPS, NGPS, and TSS had a strong and positive correlation. Flag leaf senescence fell in the negative quadrant of PCA, indicating this trait contributed to reducing the grain yield of wheat genotypes (Figure 4A). The first principle component analysis (PC 1) covered maximum variation (95.3%), followed by the second principal component (PC 2) which covered 2.8% of the total variation. The W, X, Y, and Z dots in the PAC matrix show the ambient environment while A, B, C, and D show 8 days heat stress and 1, 2, 3, and 4 dots show 4 days of heat stress. The W, X, Y, and Z dots are scattered closely and away from the vertical line representing the close and positive performance of studied parameters under an ambient environment. The A, B, C, and D dots are scattered close to the vertical line indicating that the studied parameter are not closely related and senescence has strong and negative relation with the studied parameters. The 1, 2, 3, and 4 dots of 4 days of heat stress are located away from the vertical line, indicating the close and positive performance of studied parameters than the 8 days of heat stress (Figure 4B). The maximum variation of PC 1 shows that there is a strong and negative correlation of flag leaf senescence with NGPS, GWPS and 1,000 grain weight.

**FIGURE 5:**
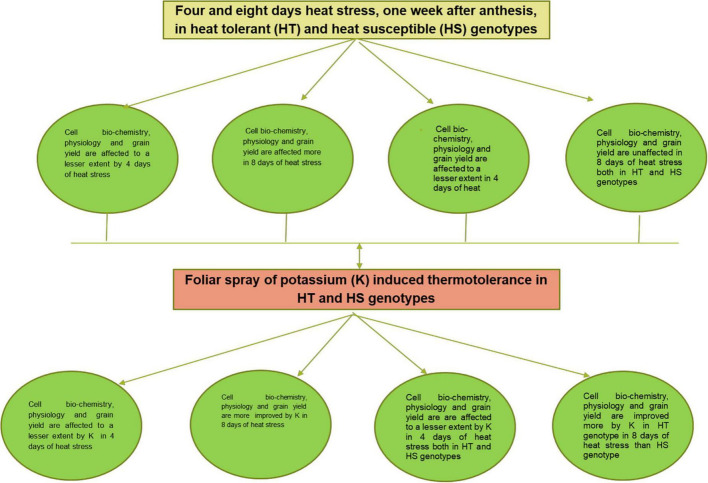
Flow diagram for the effects of post-anthesis heat stress on wheat crop and the role of foliar spray of potassium for inducing thermotolerance in wheat.

**FIGURE 6:**
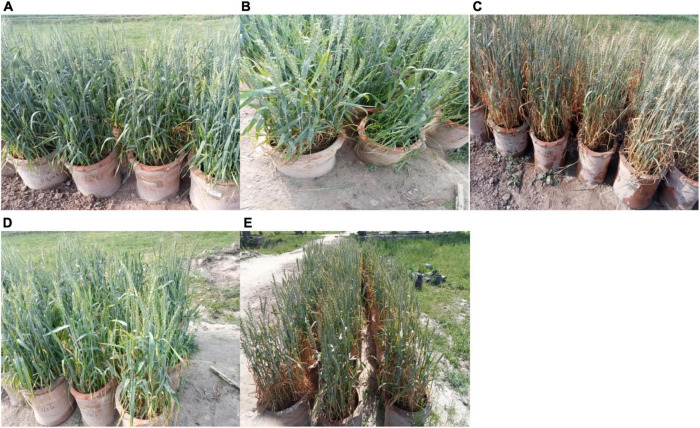
Pictorial view of wheat crop under **(A)** ambient environment post anthesis **(B)** 4 days and **(C)** 8 days heat stress **(D)** foliar spray of potassium in post anthesis 4 days, and **(E)** 8 days heat stress.

Summary of results is depicted schematically in Figures 5, 6 shows a pictorial view of wheat plants under optimal, 4 and 8 days of heat stress conditions, as well as the effects of potassium under 4 and 8 days of heat stress conditions.

## Discussions

### Impact of heat stress on yield and yield attributes

Post-anthesis initial grains development and grain filling in wheat crops are highly sensitive to short and long-duration heat spells in most parts of the world (Farooq et al., 2011; Amanullah and Fahad, 2018; Poudel et al., 2020). One of the main objectives of this study was to quantify the grain yield and weight loss in wheat 1 week after anthesis in response to 4 and 8 days of heat stress. In the current study, reduced grain yield and flag leaf senescence were strongly correlated to post-flowering (1 week after anthesis) heat 32–33°C. This period conoids the initial phase of grains development in wheat crops, which is highly sensitive to abiotic stresses (Stone and Nicolas, 1995). Short-term (3–4 days) heat stress (35–40°C) after anthesis significantly reduces yield (23%) and yield attributes in wheat (Stone and Nicholas, 1994; Wardlaw and Wrigley, 1994). Post anthesis high temperature (>31°C) diminishes the grain filling period in wheat (Wardlaw and Moncur, 1995). High temperature (31/20°C) during grain filling reduces grain weight in wheat (Dias and Lidon, 2009). Post-anthesis heat could stimulate flag leaf senescence, aborts initial small grains and affects the grain filling process, which directly reduces the final grain yield in wheat (Talukder et al., 2014). At post-anthesis in wheat, flag leaf senescence starts, while short and long-duration heat spells at this stage trigger the production of stress hormones (Hasanuzzaman et al., 2013), which restrict the translocation of carbohydrates toward developing grains (Feng et al., 2014). For example, heat stress in developing wheat kernels affects the starch synthase and reduces starch accumulation by 65% (Barnabás et al., 2008; Zahra et al., 2021). Heat stress in wheat 3–7 days after anthesis reduces grain numbers per spike and 1,000 grain weight, possibly due to abortion of young grains and an increase in photorespiration (Mohammadi et al., 2004; Marcela et al., 2017). In the current study, compared with control (ambient), the plants exposed to 4 and 8 hot days in the second year produced 11 and 19% fewer grains per spike, respectively (averaged across the genotypes). Similarly, 1,000 grain weight was also reduced by 14 and 37% in response to 4 and 8 hot days of heat stress, respectively. This loss in grain yield could be attributed to heat-induced ethylene production, decreased photosystem efficiency, and increased oxidative stress, which increased flag leaf senescence (Hays et al., 2007; Narayanan, 2018; Atif et al., 2021). The initial phase of grain formation in wheat is most sensitive to heat, and a heat shock during this period could abort grains (Fan et al., 2018). Bergkamp et al. (2018) documented that post-anthesis heat stress shortens the grain filling duration and restricts resource allocation toward developing grains. Post-anthesis heat spells in wheat damaged the leaf physiology and grain weight more in heat sensitive genotype than heat tolerant genotype (Dhyani et al., 2013; Mirosavljević et al., 2021). The better performance of heat tolerant genotype under short and long duration heat spells of present study could be associated with better cell bio-chemistry, membrane stability, sustained of leaf physiology and grain yield (Rehman et al., 2016; Yadav et al., 2018; Rajametov et al., 2021).

Post-anthesis heat in wheat affects photosynthesis, chlorophyll contents, and thylakoid membranes, triggering leaf senescence (Blum, 1998). In this study, significantly Pn, Fv/Fm were reduced by 15 and 16% in 4 days, while 37 and 36% reduction were observed in 8 days of heat stress (across the genotypes and first year of study). A short episode of heat (3 days of heat stress) at grain filling can reduce Pn and Fv/Fm of wheat crops by 69.9 and 41.16%, as suggested by Feng et al. (2014). Accumulation and translocation of water-soluble carbohydrates toward developing grains contribute to grain size during grain filling (Talukder et al., 2013), and translocation of water-soluble carbohydrates in wheat is highly sensitive to heat stress (Fan et al., 2018). Post-anthesis oxidative injury can significantly impair carbohydrate translocation to developing wheat grains (Yin et al., 2008). Although stem soluble carbohydrates were increased by 54% in the current study (across the heat spells and genotypes), this increase was not sufficient to sustain the source-sink relationship (Ovenden et al., 2017), potentially due to the inactivation of key enzymes regulating carbohydrate translocation from stem to grains (Zhao et al., 2008).

In this study, high temperature at post-anthesis significantly accelerated the oxidative stress, as evidenced by increased MDA (63% in the second year of study across the genotypes) of 8 days of heat-stressed leaves. Although the heat-stressed plants in this study upregulated enzymatic antioxidants, the increase was insufficient to maintain a balance between the plant defensive system and oxidative stress, leading to an increase in membrane damage (Sarwar et al., 2021). The high-temperature stress induces membrane damage in wheat and cotton due to higher lipid peroxidation of membranes (Savicka and Škute, 2010). The superior performance of the tolerant genotype (Ujala-16) in this study is associated with its capacity to sustain membrane stability and cell physiology. For example, the heat-tolerant genotype of this study produced 23% more grain yield than the heat susceptible genotype. This provides evidence that to combat high-temperature stress in wheat at post-anthesis, the development of heat-tolerant genotypes based on membrane stability, leaf physiology, and cell biochemistry could be a suitable technique, supports of initial hypothesis and objectives. Farheen et al. (2021) documented that heat tolerant genotypes in wheat produce higher seed yield under high-temperature stress.

### K protects wheat crops from heat damage by restraining leaf physiology

Potassium is an important plant nutrient and has a role in heat stress in maintaining photosynthesis, translocation of water-soluble carbohydrates and stimulating the plant defensive system (Hasanuzzaman et al., 2018). Exogenous application of K and higher leaf concentration of K improve heat stress and salt stress tolerance in maize and wheat under field and controlled conditions (Abbasi et al., 2014; Shahid et al., 2019). We found good support for our hypothesis that foliar spray of K alleviated the adverse effects of heat stress on wheat grain yield. Importantly, exogenous application of K increased grain numbers per spike (12%), 1,000 grain weight (19%) and also reduced flag leaf senescence (27%) in heat-stressed plants of wheat under 8 days of heat in the second years of study (across the genotype). The increase in grain numbers could be associated with restricted ethylene production, which reduced the abscission of peduncles and abortion of newly developed grains in spikelets (Ruan et al., 2012; Ali et al., 2017). Exogenous application of K in wheat at post-anthesis could improve the stay green character associated with synthesizing cytokinin and carbohydrates (Luo et al., 2019). Applying K in wheat under abiotic stress has increased the stay green character by reducing oxidative stress (Beaton and Sekhon, 1985). The increase in grain number and grain yield in wheat due to exogenous application of K is associated with high membrane stability, photosynthetic efficiency, carbohydrates accumulation and translocation toward developing grains (Jan et al., 2017). Potassium application to post-anthesis stressed wheat increases grain yield and weight by 35 and 48% could be due to the higher photosynthetic efficiency of wheat leaves (Aown et al., 2012). The increase in grain yield in wheat by exogenous application of K under drought stress has been associated with the activation of photosynthetic enzymes, maintenance of water relations, and translocation of metabolites toward developing grains (Lv et al., 2017). This implies that the foliar spray of K before the onset of stress would help increase heat tolerance in wheat.

The current study suggested that heat tolerant genotype response is relatively stronger to K application than heat susceptible genotype. Exogenous application of K improves further thermotolerance in heat tolerant genotype than heat susceptible genotype due to increased membrane stability, stimulation of antioxidants and plant defensive genes in heat tolerant genotype (Kumar et al., 2012; Rani et al., 2013; Fahad et al., 2014). Approximately 16 and 22% more grains per plant and 1,000 grain weight were observed due to K application in heat tolerant genotype than heat susceptible genotype in 8 days of heat stress in the second year of study. Our results suggested that K applied during heat stress (4 or 8 days) sustained leaf photosynthesis and significantly protected the cell membrane from damage (RCI and MDA contents). For example, Pn, Gs, Fv/Fm, and Chl a of K_–_treated leaves were increased by 22, 23, 25, and 20% increase was observed in 8 days of heat stress in the second year of study compared with water-treated leaves of respective hot environments (averaged across the genotypes). In our study, the recovery in net photosynthetic rate and efficiency of heat-stressed wheat under K application could be associated with an increased accumulation of osmoprotectants (Marschner, 1995) and protected membranes from oxidative stress (Fahad et al., 2013; Lv et al., 2017; Al-Zahrani et al., 2022). Exogenous application of K stimulates the production of antioxidants and improves chlorophyll contents and the photosynthetic rate in leaves of cotton and flower plants (Egilla et al., 2001; Zahoor et al., 2017). In heat tolerant genotype, K improved thermotolerance by strengthening the leaf physiology and membrane stability (Xu et al., 2011). This implies that exogenous application of K in heat tolerant wheat genotypes could further improve leaf physiology and membrane stability, representing a good indicator of heat tolerance (Dias and Lidon, 2010; Shahid et al., 2019) and supports our initial hypothesis and objectives.

Total water-soluble sugars (TSS) and enzymatic antioxidants (SOD, POD, and CAT) were increased by 54, 85, 90, and 78%, respectively, in the current study over the water-treated plants of 8 days of heat stress (across the genotypes and in the second year of study). This increase in TSS and enzymatic antioxidants under high-temperature stress in wheat could be associated with carbohydrates synthesis, their accumulation and the stimulation of the defensive system (Fahad et al., 2017; Khan et al., 2020). The total soluble sugars and antioxidants have been found to increase under heat stress in wheat by exogenous application of potassium (Hong-Bo et al., 2006). The results of this study imply that K may be a potential plant stimulator under abiotic stresses, particularly under heat stress. The results of this study can be generalized to other crops after the detailed studies on the role of K under various abiotic stresses.

## Conclusion

Post-anthesis short duration heat (4 days) showed a mild effect, although the sustained heat (8 days) severely affected leaf physiology, plant defensive system, and grain yield of both wheat genotypes, with significantly more damage to heat susceptible genotype. Further, 8 days of post-anthesis heat caused more damage to grain weight than numbers. Foliar application of K in this study induced thermotolerance and strengthened the plant physiology and defensive system of both genotypes. The most pronounced effect of K on plant physiology and grain yield was observed in heat tolerant genotype under 8 hot days. Our study suggests that wheat crops can be protected from post-anthesis heat injury through foliar application of K before a heat spell. Although, further field experiments are needed to confirm the efficacy of K with short and long-duration heat spells.

## Data availability statement

The original contributions presented in this study are included in the article/Supplementary material, further inquiries can be directed to the corresponding author.

## Author contributions

MS, MFS, and NS designed and wrote the manuscript and helped in data recording and data analysis. HM, NU, AK, MS, MW, HZ, MT, YS, XX, and HZ reviewed and edited the manuscript. All authors have read and approved the submitted version.

## References

[B1] Abbasi G. H. Akhtar J. Anwar-ul-Haq M. Ali S. Chen Z. Malik W. (2014). Exogenous potassium differentially mitigates salt stress in tolerant and sensitive maize hybrids. *Pak. J. Bot.* 46 135–146.

[B2] Ali Q. Daud M. K. Haider M. Z. Ali S. Rizwan M. Aslam N. (2017). Seed priming by sodium nitroprusside improves salt tolerance in wheat (*Triticum aestivum* L.) by enhancing physiological and biochemical parameters. *Plant Physiol. Biochem.* 119 50–58. 10.1016/j.plaphy.2017.08.010 28843888

[B3] Al-Zahrani H. S. Alharby H. F. Fahad S. (2022). Antioxidative defense system, hormones, and metabolite accumulation in different plant parts of two contrasting rice cultivars as influenced by plant growth regulators under heat stress. *Front. Plant Sci.* 13:911846. 10.3389/fpls.2022.911846 35712584 PMC9196032

[B4] Amanullah Fahad S. (2018). *Corn – production and human health in changing climate.* London: IntechOpen. 10.5772/intechopen.74074

[B5] Aown M. Raza S. Saleem M. F. Anjum S. A. Khaliq T. Wahid M. A. (2012). Foliar application of potassium under water deficit conditions improved the growth and yield of wheat (*Triticum aestivum* L.). *J. Anim. Plant Sci.* 22 431–437.

[B6] Arnon D. I. (1949). Copper enzymes in isolated chloroplasts. polyphenoloxidase in beta vulgaris. *Plant Physiol.* 24:1. 10.1104/pp.24.1.1 16654194 PMC437905

[B7] Asseng S. Ewert F. Martre P. Rötter R. P. Lobell D. B. Cammarano D. (2015). Rising temperatures reduce global wheat production. *Nat. Clim. Chang.* 5 143–147. 10.1038/nclimate2470

[B8] Atif B. Hesham A. Fahad S. (2021). Biochar coupling with phosphorus fertilization modifies antioxidant activity, osmolyte accumulation and reactive oxygen species synthesis in the leaves and xylem sap of rice cultivars under high-temperature stress. *Physiol. Mol. Biol. Plant* 27 2083–2100. 10.1007/s12298-021-01062-7 34629780 PMC8484400

[B9] Bala P. Sikder S. (2017). Evaluation of heat tolerance of wheat genotypes through membrane thermostability test. *J. Agric. Sci.* 2 1–6. 10.22161/ijeab/2.4.50

[B10] Barber S. A. (1984). Liming materials and practices. *Soil Acid Liming* 12, 171–209. 10.2134/agronmonogr12.2ed.c4

[B11] Barnabás B. Jäger K. Fehér A. (2008). The effect of drought and heat stress on reproductive processes in cereals. *Plant Cell Environ.* 31 11–38. 17971069 10.1111/j.1365-3040.2007.01727.x

[B12] Beaton J. D. Sekhon G. S. (1985). “Potassium nutrition of wheat and other small grains,” in *Potassium in agriculture*, ed. Munson R. D. (Madison, WI: ASA-CSSA), 701–752.

[B13] Bergkamp B. Impa S. Asebedo A. Fritz A. Jagadish S. K. (2018). Prominent winter wheat varieties response to post-flowering heat stress under controlled chambers and field based heat tents. *Field Crop Res.* 222 143–152. 10.1016/j.fcr.2018.03.009

[B14] Blum A. (1998). Improving wheat grain filling under stress by stem reserve mobilization. *Euphytica* 100 77–83. 10.1023/A:1018303922482

[B15] Cakmak I. Horst W. J. (1991). Effect of aluminium on lipid peroxidation, superoxide dismutase, catalase, and peroxidase activities in root tips of soybean (*Glycine max* L.). *Physiol. Plant* 83 463–468. 10.1111/j.1399-3054.1991.tb00121.x

[B16] Chaurasiya A. Singh D. Dutta S. K. Roy A. (2018). Growth and yield enhancement of wheat through foliar spray of osmoprotectants under high temperature stress condition. *J. Pharmacogn. Phytochem.* 7 2819–2825.

[B17] Collins B. Najeeb U. Luo Q. Tan D. K. (2022). Contribution of climate models and APSIM phenological parameters to uncertainties in spring wheat simulations: Application of SUFI-2 algorithm in northeast Australia. *J. Agron. Crop Sci.* 208 225–242. 10.1111/jac.12575

[B18] Dhyani K. Ansari M. W. Rao Y. R. Verma R. S. Shukla A. Tuteja N. (2013). Comparative physiological response of wheat genotypes under terminal heat stress. *Plant Signal. Behav.* 8:e24564. 10.4161/psb.24564 23603954 PMC3906425

[B19] Dhyani V. C. Kumar R. Pandey D. S. Singh V. P. Singh I. P. Sharma Y. (2016). Improving productivity of wheat through mulching and foliar nutrition in late sown wheat. *Intern. J. Bio Res. Stress Manag.* 7 990–995. 10.23910/IJBSM/2016.7.5.1564 3698206

[B20] Dias A. S. Lidon F. C. (2010). Bread and durum wheat tolerance under heat stress: A synoptical overview. *Emir. J. Food Agric.* 22, 412–436. 10.9755/ejfa.v22i6.4660

[B21] Dias A. S. Lidon F. C. (2009). Evaluation of grain filling rate and duration in bread and durum wheat, under heat stress after anthesis. *J. Agron. Crop Sci.* 195 137–147. 10.1111/j.1439-037X.2008.00347.x

[B22] Dias A. S. Barreiro M. G. Campos P. S. Ramalho J. C. Lidon F. C. (2010). Wheat cellular membrane thermotolerance under heat stress. *J. Agron. Crop Sci.* 196 100–108. 10.1111/j.1439-037X.2009.00398.x

[B23] Djanaguiraman M. Narayanan S. Erdayani E. Prasad P. V. (2020). Effects of high temperature stress during anthesis and grain filling periods on photosynthesis, lipids and grain yield in wheat. *BMC Plant Biol.* 20:268. 10.1186/s12870-020-02479-0 32517754 PMC7285450

[B24] Egilla J. N. Davies F. T. Drew M. C. (2001). Effect of potassium on drought resistance of *Hibiscus rosa-sinensis* cv. leprechaun: Plant growth, leaf macro-and micronutrient content and root longevity. *Plant Soil* 229 213–224. 10.1023/A:1004883032383

[B25] El Sabagh A. Hossain A. Barutcular C. Islam M. S. Awan S. I. Galal A. (2019). Wheat (*Triticum aestivum* L.) production under drought and heat stress–adverse effects, mechanisms and mitigation: A review. Corvinus university of Budapest. *Appl. Ecol. Environ. Res.* 17 8307–8332. 10.15666/aeer/1704_83078332

[B26] Fahad S. Bajwa A. A. Nazir U. Anjum S. A. Farooq A. Zohaib A. (2017). Crop production under drought and heat stress: Plant responses and management options. *Front. Plant Sci.* 8:1147. 10.3389/fpls.2017.01147 28706531 PMC5489704

[B27] Fahad S. Chen Y. Saud S. Wang K. Xiong D. Chen C. (2013). Ultraviolet radiation effect on photosynthetic pigments, biochemical attributes, antioxidant enzyme activity and hormonal contents of wheat. *J Food Agric. Environ.* 11 1635–1641.

[B28] Fahad S. Hussain S. Matloob A. Khan F. A. Khaliq A. Saud S. (2014). Phytohormones and plant responses to salinity stress: A review. *Plant Growth Regul.* 75 391–404. 10.1007/s10725-014-0013-y

[B29] Fan Y. Ma C. Huang Z. Abid M. Jiang S. Dai T. (2018). Heat priming during early reproductive stages enhances thermo-tolerance to post-anthesis heat stress via improving photosynthesis and plant productivity in winter wheat (*Triticum aestivum* L.). *Front. Plant Sci.* 9:805. 10.3389/fpls.2018.00805 29951079 PMC6008404

[B30] Farheen R. Ahmad M. Q. Saleem M. A. Qayyum A. Noor E. Malik W. (2021). Genetic diversity, population structure and evaluation of bread wheat genotypes under high temperature stress. *J. Anim. Plant Sci.* 31 1015–1027.

[B31] Farooq M. Bramley H. Palta J. A. Siddique K. H. (2011). Heat stress in wheat during reproductive and grain-filling phases. *Crit. Rev. Plant Sci.* 30 491–507. 10.1080/07352689.2011.615687

[B32] Feng B. Liu P. Li G. Dong S. T. Wang F. H. Kong L. A. (2014). Effect of heat stress on the photosynthetic characteristics in flag leaves at the grain-filling stage of different heat-resistant winter wheat varieties. *J. Agron. Crop Sci.* 200 143–155. 10.1111/jac.12045

[B33] Gäth S. Meuser H. Abitz C. A. Wessolek G. Renger M. (1989). Determination of potassium delivery to the roots of cereal plants. *Z. Pflanzen. Bod.* 152 143–149. 10.1002/jpln.19891520203

[B34] Giannopolitis C. N. Ries S. K. (1977). Superoxide dismutases: I. Occurrence in higher plants. *Plant Physiol.* 59 309–314. 10.1104/pp.59.2.309 16659839 PMC542387

[B35] Gomez K. A. Gomez A. A. (1984). *Statistical procedures for agricultural research*. New York, NY: John Wiley and sons.

[B36] Hasanuzzaman M. Mahmud J. A. Anee T. I. Nahar K. Islam M. T. (2018). “Drought stress tolerance in wheat: Omics approaches in understanding and enhancing antioxidant defense,” in *Abiotic stress-mediated sensing and signaling in plants: An omics perspective*, eds Zargar S. Zargar M. (Singapore: Springer), 267–307. 10.1007/978-981-10-7479-0_10

[B37] Hasanuzzaman M. Nahar K. Alam M. M. Roychowdhury R. Fujita M. (2013). Physiological, biochemical, and molecular mechanisms of heat stress tolerance in plants. *Intern. J. Mol. Sci.* 14 9643–9684. 10.3390/ijms14059643 23644891 PMC3676804

[B38] Hassanein R. A. El-Khawas S. A. Ibrahim S. K. El-Bassiouny H. M. Mostafa H. A. Abdel-Monem A. A. (2013). Improving the thermo tolerance of wheat plant by foliar application of arginine or putrescine. *Pak. J. Bot.* 45 111–118.

[B39] Hays D. Mason E. Do J. H. Menz M. Reynolds M. (2007). “Expression quantitative trait loci mapping heat tolerance during reproductive development in wheat (*Triticum aestivum*),” in *Wheat production in stressed environments*, eds Buck H. T. Nisi J. E. Salomón N. (Dordrecht: Springer), 373–382. 10.1007/1-4020-5497-1_46

[B40] Hong-Bo S. Xiao-Yan C. Li-Ye C. Xi-Ning Z. Gang W. Yong-Bing Y. (2006). Investigation on the relationship of proline with wheat anti-drought under soil water deficits. *Colloid Surf. B Biointerf*. 53, 113–119. 10.1016/j.colsurfb.2006.08.008 16979325

[B41] Hu L. Zhang Z. Xiang Z. Yang Z. (2016). Exogenous application of citric acid ameliorates the adverse effect of heat stress in tall fescue (*Lolium arundinaceum* L.). *Front. Plant Sci.* 7:179. 10.3389/fpls.2016.00179 26925085 PMC4757681

[B42] IPCC (2014). *Climate change 2014: Synthesis report. Contribution of working groups I, II and III to the fifth assessment report of the intergovernmental panel on climate change.* Geneva: IPCC.

[B43] Jan A. U. Hadi F. Midrarullah N. M. A. Rahman K. (2017). Potassium and zinc increase tolerance to salt stress in wheat (*Triticum aestivum* L.). *Plant Physiol. Biochem.* 116 139–149. 10.1016/j.plaphy.2017.05.008 28558283

[B44] Jifon J. L. Lester G. E. (2009). Foliar potassium fertilization improves fruit quality of field-grown muskmelon on calcareous soils in south Texas. *J. Sci. Food Agric.* 89 2452–2460. 10.1002/jsfa.3745

[B45] Khan M. A. Shirazi M. U. Shereen A. I. S. H. A. Ali M. Asma B. H. Jilani N. S. (2020). Agronomical and physiological perspectives for identification of wheat genotypes for high temperature tolerance. *Pak. J. Bot.* 52 1973–1980. 10.30848/PJB2020-6(12)

[B46] Kumar S. Gupta D. Nayyar H. (2012). Comparative response of maize and rice genotypes to heat stress: Status of oxidative stress and antioxidants. *Acta Physiol. Plant* 34 75–86. 10.1007/s11738-011-0806-9

[B47] Lester G. E. Jifon J. L. Rogers G. (2005). Supplemental foliar potassium applications during muskmelon fruit development can improve fruit quality, ascorbic acid, and beta-carotene contents. *J. Am. Soc. Hortic. Sci.* 130 649–653. 10.21273/JASHS.130.4.649 35581909

[B48] Liu D. Zou J. Meng Q. Zou J. Jiang W. (2009). Uptake and accumulation and oxidative stress in garlic (*Allium sativum* L.) under lead phytotoxicity. *Ecotoxicology* 18 134–143. 10.1007/s10646-008-0266-1 18773294

[B49] Luo J. Wei B. Han J. Liao Y. Liu Y. (2019). Spermidine increases the sucrose content in inferior grain of wheat and thereby promotes its grain filling. *Front. Plant Sci.* 10:1309. 10.3389/fpls.2019.01309 31824519 PMC6881305

[B50] Lv X. Li T. Wen X. Liao Y. Liu Y. (2017). Effect of potassium foliage application post-anthesis on grain filling of wheat under drought stress. *Field Crop Res.* 206 95–105. 10.1016/j.fcr.2017.02.015

[B51] Marcela H. Karel K. Pavlína S. Petr Š Petr H. Kateřina N. (2017). Effect of heat stress at anthesis on yield formation in winter wheat. *Plant Soil Environ.* 63 139–144. 10.17221/73/2017-PSE 27203573

[B52] Marschner H. (1995). *Mineral nutrition of higher plants*, 2nd Edn. London: Academic Press.

[B53] Matías J. Rodríguez M. J. Cruz V. Calvo P. Reguera M. (2021). Heat stress lowers yields, alters nutrient uptake and changes seed quality in quinoa grown under Mediterranean field conditions. *J. Agron. Crop Sci.* 207 481–491. 10.1111/jac.12495

[B54] Mirosavljević M. Mikić S. Župunski V. Kondić Špika A. Trkulja D. Ottosen C. O. (2021). Effects of high temperature during anthesis and grain filling on physiological characteristics of winter wheat cultivars. *J. Agron. Crop Sci.* 207 823–832. 10.1111/jac.12546

[B55] Mohammadi V. Qannadha M. R. Zali A. A. Yazdi-Samadi B. (2004). Effect of post anthesis heat stress on head traits of wheat. *Intern. J. Agric. Biol.* 6 42–44.

[B56] Mondal S. Singh R. P. Mason E. R. Huerta-Espino J. Autrique E. Joshi A. K. (2016). Grain yield, adaptation and progress in breeding for early-maturing and heat-tolerant wheat lines in South Asia. *Field Crop Res.* 192 78–85. 10.1016/j.fcr.2016.04.017 27307654 PMC4892352

[B57] Nagar S. Singh V. P. Arora A. Dhakar R. Ramakrishnan S. (2015). Assessment of terminal heat tolerance ability of wheat genotypes based on physiological traits using multivariate analysis. *Acta Physiol. Plant* 37 1–9. 10.1007/s11738-015-2017-2

[B58] Narayanan S. (2018). Effects of high temperature stress and traits associated with tolerance in wheat. *Open Access J. Sci*. 2, 177–186. 10.15406/oajs.2018.02.00067

[B59] Ovenden B. Milgate A. Lisle C. Wade L. J. Rebetzke G. J. Holland J. B. (2017). Selection for water-soluble carbohydrate accumulation and investigation of genetic× environment interactions in an elite wheat breeding population. *Theor. Appl. Genet.* 130 2445–2461. 10.1007/s00122-017-2969-2 28852799

[B60] Pei Z. M. Murata Y. Benning G. Thomine S. Sener B. K. Allen G. J. (2000). Calcium channels activated by hydrogen peroxide mediate abscisic acid signalling in guard cells. *Nature* 406, 731–734. 10.1038/35021067 10963598

[B61] Porter J. R. Gawith M. (1999). Temperatures and the growth and development of wheat: A review. *Eur. J. Agron.* 10 23–36. 10.1016/S1161-0301(98)00047-1

[B62] Poudel M. R. Ghimire S. Dhakal K. H. Thapa D. B. Poudel H. K. (2020). Evaluation of wheat genotypes under irrigated, heat stress and drought conditions. *J. Biol. Todays World* 9 1–12.

[B63] Prasad P. V. V. Staggenborg S. A. Ristic Z. (2008). “Impacts of drought and/or heat stress on physiological, developmental, growth, and yield processes of crop plants,” in *Response of crops to limited water: Understanding and modeling water stress effects on plant growth processes. Advances in agricultural systems modeling series 1*, eds Ahuja L. H. Saseendran S. A. (Madison, WI: ASA-CSSA), 301–355.

[B64] Raghunath M. Beena R. Mohan V. Viji M. Manju R. Stephen R. (2021). High temperature stress mitigation in rice (*Oryza sativa* L.): Foliar application of plant growth regulators and nutrients. *J Crop Weed* 17 34–47. 10.22271/09746315.2021.v17.i1.1404

[B65] Rahman A. Rahman M. M. Hasan M. M. Begum F. Sarker M. A. Z. (2014). Effects of foliar application of potassium orthophosphate on grain yield and kernel quality of Wheat (*Triticum aestivum* L.) under terminal heat stress. *Bangladesh J. Agric. Res.* 39 67–77. 10.3329/bjar.v39i1.20144

[B66] Rajametov S. N. Yang E. Y. Cho M. C. Chae S. Y. Jeong H. B. Chae W. B. (2021). Heat-tolerant hot pepper exhibits constant photosynthesis via increased transpiration rate, high proline content and fast recovery in heat stress condition. *Sci. Rep.* 11 1–9. 10.1038/s41598-021-93697-5 34253784 PMC8275607

[B67] Rani P. L. Sreenivas G. Reddy D. R. Rao V. P. Surekha K. Sankar A. S. (2013). Influence of dates of sowing and nitrogen levels on growth and yield of Kharif maize under irrigated conditions in South Telanagana Agro-climatic Zone of Andhra Pradesh, India. *Intern. J. Bio Res. Stress Manag.* 4 34–42.

[B68] Rehman S. U. Bilal M. Rana R. M. Tahir M. N. Shah M. K. N. Ayalew H. (2016). Cell membrane stability and chlorophyll content variation in wheat (*Triticum aestivum*) genotypes under conditions of heat and drought. *Crop Pasture Sci.* 67 712–718. 10.1071/CP15385

[B69] Ruan Y. L. Patrick J. W. Bouzayen M. Osorio S. Fernie A. R. (2012). Molecular regulation of seed and fruit set. *Trend Plant Sci.* 17 656–665. 10.1016/j.tplants.2012.06.005 22776090

[B70] Saeed U. Dempewolf J. Becker-Reshef I. Khan A. Ahmad A. Wajid S. A. (2017). Forecasting wheat yield from weather data and MODIS NDVI using random forests for Punjab province, Pakistan. *Intern. J. Remote Sens.* 38 4831–4854. 10.1080/01431161.2017.1323282

[B71] Saleem M. F. Kamal M. A. Anjum S. A. Shahid M. Raza M. A. S. Awais M. (2018). Improving the performance of Bt-cotton under heat stress by foliar application of selenium. *J. Plant Nutr.* 41, 1711–1723. 10.1080/01904167.2018.1459694

[B72] Sarwar M. Saleem M. F. Ali B. Nadeem M. Ghani M. A. Zhou W. (2021). Improving thermotolerance in *Gossypium hirsutum* by using signalling and non-signalling molecules under glass house and field conditions. *Indust. Crop Prod.* 172:113996. 10.1016/j.indcrop.2021.113996

[B73] Sarwar M. Saleem M. F. Najeeb U. Shakeel A. Ali S. Bilal M. F. (2017). Hydrogen peroxide reduces heat-induced yield losses in cotton (*Gossypium hirsutum* L.) by protecting cellular membrane damage. *J. Agron. Crop Sci.* 203 429–441. 10.1111/jac.12203

[B74] Sarwar M. Saleem M. F. Ullah N. Ali S. Rizwan M. Shahid M. R. (2019). Role of mineral nutrition in alleviation of heat stress in cotton plants grown in glasshouse and field conditions. *Sci. Rep.* 9 1–17. 10.1038/s41598-019-49404-6 31506449 PMC6737086

[B75] Sarwar M. Saleem M. F. Ullah N. Rizwan M. Ali S. Shahid M. R. (2018). Exogenously applied growth regulators protect the cotton crop from heat-induced injury by modulating plant defense mechanism. *Sci. Rep.* 8 1–15. 10.1038/s41598-018-35420-5 30459328 PMC6244283

[B76] Savicka M. Škute N. (2010). Effects of high temperature on malondialdehyde content, superoxide production and growth changes in wheat seedlings (*Triticum aestivum* L.). *Ekologija* 56 26–33. 10.2478/v10055-010-0004-x

[B77] Shahid M. Saleem M. F. Saleem A. Raza M. A. S. Kashif M. Shakoor A. (2019). Exogenous potassium–instigated biochemical regulations confer terminal heat tolerance in wheat. *J. Soil Sci. Plant Nutr.* 19 137–147. 10.1007/s42729-019-00020-3

[B78] Shahid M. Saleem M. F. Saleem A. Sarwar M. Khan H. Z. Shakoor A. (2020). Foliar potassium-induced regulations in glycine betaine and malondialdehyde were associated with grain yield of heat-stressed bread wheat (*Triticum aestivum* L.). *J. Soil Sci. Plant Nutr.* 20 1785–1798. 10.1007/s42729-020-00250-w

[B79] Sharkey T. D. (2005). Effects of moderate heat stress on photosynthesis: Importance of thylakoid reactions, rubisco deactivation, reactive oxygen species, and thermotolerance provided by isoprene. *Plant Cell Environ.* 28 269–277. 10.1111/j.1365-3040.2005.01324.x

[B80] Shenoda J. E. Sanad M. N. Rizkalla A. A. El-Assal S. Ali R. T. Hussein M. H. (2021). Effect of long-term heat stress on grain yield, pollen grain viability and germinability in bread wheat (*Triticum aestivum* L.) under field conditions. *Heliyon* 7:e07096. 10.1016/j.heliyon.2021.e07096 34141912 PMC8187965

[B81] Shin R. Schachtman D. P. (2004). Hydrogen peroxide mediates plant root cell response to nutrient deprivation. *Proc. Natl. Acad. Sci. U.S.A.* 101 8827–8832. 10.1073/pnas.0401707101 15173595 PMC423280

[B82] Siddique A. Kandpal G. Kumar P. (2018). Proline accumulation and its defensive role under diverse stress condition in plants: An overview. *J. Pure Appl. Microbiol.* 12 1655–1659. 10.22207/JPAM.12.3.73

[B83] Steel R. G. D. Torrie J. H. (1960). *Principles and procedures of statistics. Principles and procedures of statistics*. New York, NY: McGraw-Hill Book Co.

[B84] Stone P. J. Nicholas M. E. (1994). Wheat cultivars vary widely in their responses of grain yield and quality to short periods of post-anthesis heat stress. *Funct. Plant Biol.* 21 887–900. 10.1071/PP9940887

[B85] Stone P. J. Nicolas M. E. (1995). Effect of timing of heat stress during grain filling on two wheat varieties differing in heat tolerance. I. Grain growth. *Funct. Plant Biol.* 22 927–934. 10.1071/PP9950927

[B86] Sullivan C. Y. (1972). “Mechanisms of heat and drought resistance in grain sorghum and methods of measurement,” in *Sorghum in seventies*, eds Rao N. House L. (New Delhi: Oxford and IBHPublishing Co), 248–264.

[B87] Sun C. Lu L. Liu L. Liu W. Yu Y. Liu X. (2014). Nitrate reductase-mediated early nitric oxide burst alleviates oxidative damage induced by aluminum through enhancement of antioxidant defenses in roots of wheat (*Triticum aestivum* L.). *New phytol.* 201 1240–1250. 10.1111/nph.12597 24237306

[B88] Talukder A. S. M. H. M. McDonald G. K. Gill G. S. (2013). Effect of short-term heat stress prior to flowering and at early grain set on the utilization of water-soluble carbohydrate by wheat genotypes. *Field Crop Res.* 147 1–11. 10.1016/j.fcr.2013.03.013

[B89] Talukder A. S. M. H. M. McDonald G. K. Gill G. S. (2014). Effect of short-term heat stress prior to flowering and early grain set on the grain yield of wheat. *Field Crop Res.* 160 54–63. 10.1016/j.fcr.2014.01.013

[B90] Ullah N. Ababaei B. Chenu K. (2020). Increasing heat tolerance in wheat to counteract recent and projected increases in heat stress. *Multidiscip. Digit. Publ. Inst. Proc.* 36:132. 10.3390/proceedings2019036132

[B91] Ullah N. Chenu K. (2019). “Impact of post-flowering heat stress on staygreen and grain development in wheat,” in *Proceeedings of the Agronomy Australia Conference*, Wagga Wagga, NSW, 25–29.

[B92] Ullah N. Tan D. K. Y. Sarwar M. Ali S. (2019). “Adaptation of crops to warmer climates: Morphological and physiological mechanisms,” in *Sustainable solutions for food security*, eds Sarkar A. Sensarma S. vanLoon G. (Cham: Springer), 27–50. 10.1007/978-3-319-77878-5_2

[B93] Waraich E. A. Ahmad R. Halim A. Aziz T. (2012). Alleviation of temperature stress by nutrient management in crop plants: A review. *J. Soil Sci. Plant Nutr.* 12 221–244. 10.4067/S0718-95162012000200003 27315006

[B94] Wardlaw I. F. Moncur L. (1995). The response of wheat to high temperature following anthesis. I. The rate and duration of kernel filling. *Funct. Plant Biol.* 22 391–397. 10.1071/PP9950391

[B95] Wardlaw I. F. Wrigley C. W. (1994). Heat tolerance in temperate cereals: An overview. *Funct. Plant Biol.* 21 695–703. 10.1071/PP9940695 26340626

[B96] Wells K. L. Murdock L. W. Dougherty C. T. (1982). *Fertilization of cool season grasses*. Lexington, KY: University of Kentucky College of agriculture cooperative extension service AGR-103.

[B97] Xu G. Zhang F. Shah S. G. Ye Y. Mao H. (2011). Use of leaf color images to identify nitrogen and potassium deficient tomatoes. *Pattern Rec. Letter.* 32 1584–1590. 10.1016/j.patrec.2011.04.020

[B98] Yadav S. K. Tiwari Y. K. Singh V. Patil A. A. Shanker A. K. Jyothi Lakshmi N. (2018). Physiological and biochemical basis of extended and sudden heat stress tolerance in maize. *Proc. Natl. Acad. Sci. India Sect. B Biol. Sci.* 88 249–263. 10.1007/s40011-016-0752-9

[B99] Yemm E. W. Willis A. J. (1954). The estimation of carbohydrates in plant extracts by anthrone. *Biochem. J.* 57 508–514. 10.1042/bj0570508 13181867 PMC1269789

[B100] Yin X. L. Jiang L. Song N. H. Yang H. (2008). Toxic reactivity of wheat (*Triticum aestivum*) plants to herbicide isoproturon. *J. Agric. Food Chem.* 56 4825–4831. 10.1021/jf800795v 18522406

[B101] Zahoor R. Dong H. Abid M. Zhao W. Wang Y. Zhou Z. (2017). Potassium fertilizer improves drought stress alleviation potential in cotton by enhancing photosynthesis and carbohydrate metabolism. *Environ. Exp. Bot.* 137 73–81. 10.1016/j.envexpbot.2017.02.002

[B102] Zahra N. Wahid A. Hafeez M. B. Ullah A. Siddique K. H. Farooq M. (2021). Grain development in wheat under combined heat and drought stress: Plant responses and management. *Environ. Exper. Bot.* 188:104517. 10.1016/j.envexpbot.2021.104517

[B103] Zhao H. Dai T. Jiang D. Cao W. (2008). Effects of high temperature on key enzymes involved in starch and protein formation in grains of two wheat cultivars. *J. Agron. Crop Sci.* 194 47–54. 10.1111/j.1439-037X.2007.00283.x

