# Supplementary material for: Strengthening leaf physiological functioning and grain yield formation in heat-stressed wheat through potassium application
Source: Front Plant Sci. 2022 Oct 5;13:1005773. doi: 10.3389/fpls.2022.1005773 (PMC9611777; doi:10.3389/fpls.2022.1005773)
Supplement: Supplementary file 1 [file Table_2.DOCX]

**Table S1.** Ambient and polythean sheet maximum temperatures one week after anthesis in wheat (a) during 2018-19 (b) during 2019-20

| **(a)** | | | | **(b)** | | | |
| --- | --- | --- | --- | --- | --- | --- | --- |
| **Days of the year** | **Tmax. (°C) Ambient** | **Heat stress duration one week after anthesis** | **Tmax. (°C) of polythean sheet** | **Days of the year** | **Tmax. (°C) Ambient** | **Heat stress duration one week after anthesis** | **Tmax. (°C) of polythean sheet** |
| 27-Feb | 28 | 4 days | 31 ± 2 | 25-Feb | 30 | 4 days | 33 ± 2 |
| 28-Feb | 28 |  | 31 ± 2 | 26-Feb | 30 |  | 33 ± 2 |
| 29-Feb | 23 |  | 26 ± 2 | 27-Feb | 27 |  | 30 ± 2 |
| 1-Mar | 24 |  | 27 ± 2 | 28-Feb | 28 |  | 31 ± 2 |
| 27-Feb | 28 | 8 days | 31 ± 2 | 25-Feb | 30 | 8 days | 33 ± 2 |
| 28-Feb | 28 |  | 31 ± 2 | 26-Feb | 30 |  | 33 ± 2 |
| 29-Feb | 23 |  | 26 ± 2 | 27-Feb | 27 |  | 30 ± 2 |
| 1-Mar | 24 |  | 27 ± 2 | 28-Feb | 28 |  | 31 ± 2 |
| 2-Mar | 26 |  | 29 ± 2 | 1-Mar | 29 |  | 32 ± 2 |
| 3-Mar | 27 |  | 30 ± 2 | 2-Mar | 29 |  | 32 ± 2 |
| 4-Mar | 23 |  | 26 ± 2 | 3-Mar | 29 |  | 32 ± 2 |
| 5-Mar | 22 |  | 25 ± 2 | 4-Mar | 30 |  | 33 ± 2 |

Tmax (a) mean max. temperature for 4 days (29°C) and 8 days of heat stress (28°C) under polythene sheett in 2019

Tmax (b) mean max. temperature for 4 days (32°C) and 8 days of heat stress (33°C) under polythene sheet in 2020
